# A MaxEnt Model of Citrus Black Fly *Aleurocanthus woglumi* Ashby (Hemiptera: Aleyrodidae) under Different Climate Change Scenarios

**DOI:** 10.3390/plants13040535

**Published:** 2024-02-15

**Authors:** Nilson Rodrigues da Silva, Philipe Guilherme Corcino Souza, Gildriano Soares de Oliveira, Alisson da Silva Santana, Leandro Bacci, Gerson Adriano Silva, Edmond Joseph Djibril Victor Barry, Fernanda de Aguiar Coelho, Marcus Alvarenga Soares, Marcelo Coutinho Picanço, Renato Almeida Sarmento, Ricardo Siqueira da Silva

**Affiliations:** 1 Departamento de Engenharia Agronômica do Sertão (DEAS), Universidade Federal de Sergipe (UFS), Rodovia Eng. Jorge Neto—Km 03, s/n, Nossa Senhora da Glória 49680-000, SE, Brazil; nilsonufv@gmail.com; 2 Departamento de Agronomia, Instituto Federal de Ciência e Tecnologia do Triângulo Mineiro (IFTM Campus Uberlândia), Uberlândia 38400-970, MG, Brazil; philipe.corcino@gmail.com; 3 Programa de Pós Graduação em Produção Vegetal, Universidade Federal dos Vales Jequitinhonha e Mucuri, Campus JK, Diamantina 39100-000, MG, Brazil; gildriano.oliveira@ufvjm.edu.br (G.S.d.O.); edmond.barry@ufvjm.edu.br (E.J.D.V.B.); marcus.alvarenga@ufvjm.edu.br (M.A.S.); 4 Departamento de Engenharia Agronômica (DEA), Universidade Federal de Sergipe (UFS), São Cristóvão 49100-000, SE, Brazil; alisson.da-silva-santana@unl.edu (A.d.S.S.); bacci.ufs@gmail.com (L.B.); 5 Laboratório de Entomologia e Fitopatologia, Universidade Estadual Norte Fluminense Darcy Ribeiro (UENF), Campos dos Goytacazes 28013-602, RJ, Brazil; silva.gersonadriano@gmail.com; 6 Programa de Pós-Graduação em Ciência Florestal, Universidade Federal dos Vales Jequitinhonha e Mucuri, Campus JK, Diamantina 39100-000, MG, Brazil; aguiar.fernanda@ufvjm.edu.br; 7 Departamento de Entomologia, Universidade Federal de Viçosa, Campus UFV, Viçosa 36570-000, MG, Brazil; picanco@ufv.br; 8 Programa de Pós-Graduação em Produção Vegetal, Universidade Federal do Tocantins, Campus Gurupi, Gurupi 77402-970, TO, Brazil; rsarmento@mail.uft.edu.br

**Keywords:** citrus, climatic factors, ecological niche model, climate change

## Abstract

The citrus blackfly (CBF), *Aleurocanthus woglumi* Ashby, is an exotic pest native to Southeast Asia that has spread rapidly to the world’s main centers of citrus production, having been recently introduced to Brazil. In this study, a maximum entropy niche model (MaxEnt) was used to predict the potential worldwide distribution of CBF under current and future climate change scenarios for 2030 and 2050. These future scenarios came from the Coupled Model Intercomparison Project Phase 6 (CMIP6), SSP1-2.6, and SSP5-8.5. The MaxEnt model predicted the potential distribution of CBF with area under receiver operator curve (AUC) values of 0.953 and 0.930 in the initial and final models, respectively. The average temperature of the coldest quarter months, precipitation of the rainiest month, isothermality, and precipitation of the driest month were the strongest predictors of CBF distribution, with contributions of 36.7%, 14.7%, 13.2%, and 10.2%, respectively. The model based on the current time conditions predicted that suitable areas for the potential occurrence of CBF, including countries such as Brazil, China, the European Union, the USA, Egypt, Turkey, and Morocco, are located in tropical and subtropical regions. Models from SSP1-2.6 (2030 and 2050) and SSP5-8.5 (2030) predicted that suitable habitats for CBF are increasing dramatically worldwide under future climate change scenarios, particularly in areas located in the southern US, southern Europe, North Africa, South China, and part of Australia. On the other hand, the SSP5-8.5 model of 2050 indicated a great retraction of the areas suitable for CBF located in the tropical region, with an emphasis on countries such as Brazil, Colombia, Venezuela, and India. In general, the CMIP6 models predicted greater risks of invasion and dissemination of CBF until 2030 and 2050 in the southern regions of the USA, European Union, and China, which are some of the world’s largest orange producers. Knowledge of the current situation and future propagation paths of the pest serve as tools to improve the strategic government policies employed in CBF’s regulation, commercialization, inspection, combat, and phytosanitary management.

## 1. Introduction

The citrus blackfly, *Aleurocanthus woglumi* Ashby (CBF), is a phytophagous insect native to Southeast Asia, which has spread rapidly to the southeast of the African continent and countries of South, Central, and North America [1,2]. Historically, the first CBF infestations were first observed in India (Asia) in 1910 [3], Jamaica in 1913 (Central America) [4], Florida in 1934 (North America) [5], Mexico in 1940 (North America) [5], and near Durban (Republic of South Africa) in 1959 [6]. In Brazil, the first records were observed in the State of Pará in 2001, followed by observations of spread in the states of Maranhão, Amazonas, Roraima, Amapá, Tocantins, Paraíba, Goiás, and São Paulo [7,8]. Currently, CBF is classified as an A2 quarantine pest in Brazil because it is not yet widely disseminated and, therefore, is under official control.

Due to the importance of citrus farming in the Brazilian economy, the spread of CBF to other states worries producers and government agencies linked to agriculture due to the direct and indirect damage caused by this insect pest. Direct damage occurs during feeding, when the insect inserts the stylet into the phloem of the plant leaf and promotes the continuous suction of nutrients and the injection of toxins, causing weakening and a reduction of up to 80% in the fruit set of the attacked plants [8,9]. Indirect damage, on the other hand, occurs through the elimination of sugary excretions (“honeydew”) during feeding, which favor the development of fungi of the genus *Capnodium* (“fumagina”) on the surface of leaves and fruits. Sooty mold’s partial or total covering of leaves, fruits, and branches negatively affects the plant’s physiological processes of photosynthesis, respiration, and transpiration, causing qualitative deterioration in fruit production [4]. In addition to citrus fruits, CBF can attack more than 300 species of plants, including cultivated plants, ornamentals, and weeds [10], making it difficult to control and monitor the population dynamics of this insect pest in the field.

Abiotic factors such as temperature, precipitation, and relative humidity are climatic variables that directly affect the infestation potential [11], life cycle [12], reproduction, and survival [13] of CBF in different agroecosystems. Identifying potential CBF distribution areas is a fundamental step in formulating strategies that hinder and limit the expansion of this invasive pest in other climatic conditions. In this context, ecological niche models are tools that make it possible to study the implications of climate on the distribution of insect pest populations and cultivated plants [14] in current and future scenarios. The maximum entropy model (MaxEnt) stands out for its ability to predict habitat suitability based on machine learning algorithms using correlations between data from species occurrence records and environmental variables [15]. MaxEnt has been employed as a powerful tool to project the distribution of several species, including *Dalbulus maidis* [16], *Spodoptera frugiperda* [17,18], *Ceratocystis fimbriata* [19], *Aceria guerreronis* [20], and *Bemisia tabaci* [21]. The high capacity of the MaxEnt model to predict habitat adequacy in current and future climate change scenarios stands out mainly in the reported results of these studies to determine possible global changes that may favor the expansion, retraction, or emergence of habitat fragmentation bands and directly influence the geographic distribution of a certain species.

The future climate change scenarios are worrying and increase the uncertainties about the real impact of these changes on the potential distribution areas and the success of the strategies used to monitor and manage the invasive CBF pest. To help solve this problem, we can adapt the recent Coupled Model Intercomparison Project Phase 6 (CMIP6) models to predict the global distribution of CBF populations in contrasting future scenarios with greater accuracy, considering the impacts in the most optimistic (SSP1-2.6) and pessimistic (SSP5-8.5) scenarios of temperature increase. The SSP1-2.6 scenario is based on the rapid reduction of CO_2_ emissions by society, limiting the temperature increase to 1.5 °C by 2100, while the SSP5-8.5 represents the “business as usual” considering an increase in the pattern of CO_2_ emissions, causing an increase in the average temperature of 3.3–5.7 °C by 2100 [22]. CMIP6 models stand out for their greater projection of more substantial warming, the greater climate sensitivity of the new generation of climate models, and the new set of specifications for concentration, emission, and socioeconomic development [23].

Ecological niche studies aimed at identifying suitable habitats for CBF are a fundamental step to support the creation of public policies aimed at creating phytosanitary barriers, monitoring measures, and controlling and mitigating the populations of this invasive species in the field. Currently, the occurrence of CBF is restricted to some regions or countries, which increases the need for further studies to identify suitable habitats for this insect pest in current and future scenarios. In recent study, [24] predicted the potential suitability of CBF using the RCP8.5 model of CMIP5 in the CLIMEX 4.0 program. However, to our knowledge, no studies have yet been performed using the CMIP6 dataset to predict the global suitability of CBF under future climate change scenarios (SSP1-2.6 and SSP5-8.5). In addition, developing new habitat suitability prediction maps by MaxEnt using the CMIP6 model can bring valuable information for understanding the impacts of climate change on CBF. Thus, this study aimed to use a maximum entropy niche model (MaxEnt) to predict the potential worldwide distribution of CBF under current and future climate change scenarios for 2030 and 2050.

## 2. Results

### 2.1. Modeling Performance

**Figure 1:**
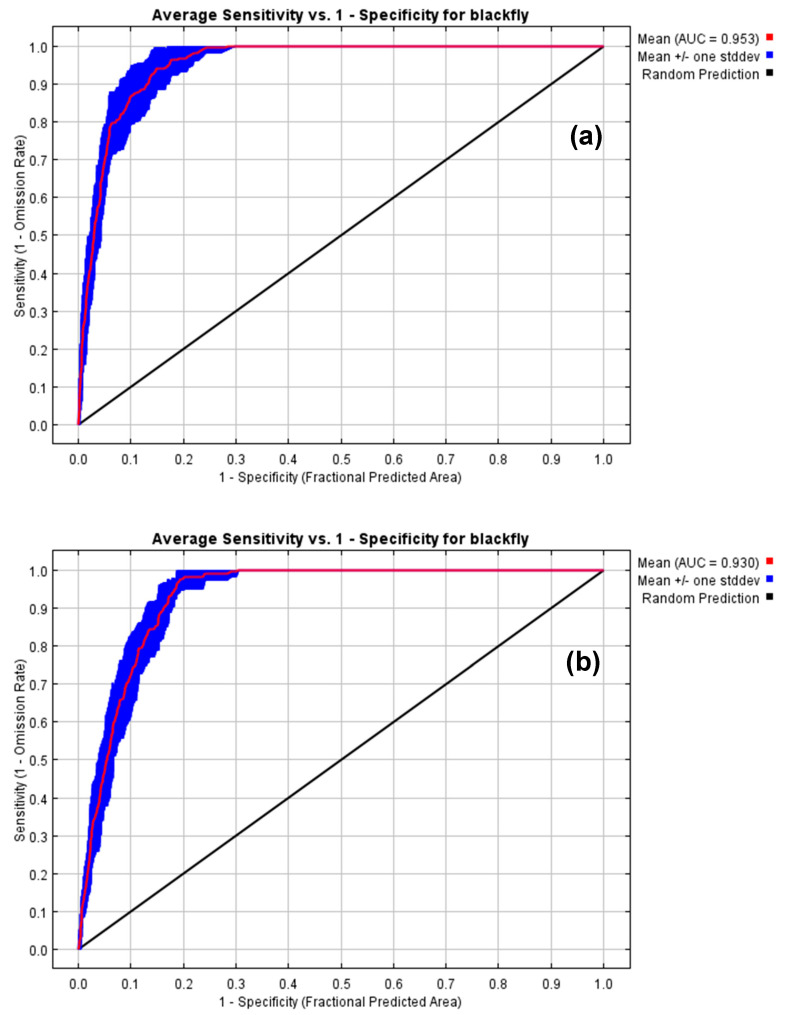
ROC curves for the initial model (**a**) and the final model (**b**).

Figure 1 shows the AUC values from 10-repeat cross-validation of the initial and final models. The values were 0.953 and 0.930, with standard deviations of 0.012 and 0.013, respectively. These results indicate the models’ lofty performance.

### 2.2. Bioclimatc Variables Contribution

Pearson correlation coefficients between the 20 bioclimatic variables are shown in Table 1, and each variable’s percentage contribution in the initial model is shown in Table 2. After applying the described method to select the best bioclimatic variables, the average temperature of the coldest quarter months (bio11), precipitation of the rainiest month (bio13), and precipitation of the driest month (bio14) were chosen for the final model. Thus, the final model for CBF was developed, and its accuracy was evaluated.

In the final model, all variables contributed similarly to the model (Table 3). Together, these variables hold a percentage contribution of 90.9%.

**Figure 2:**
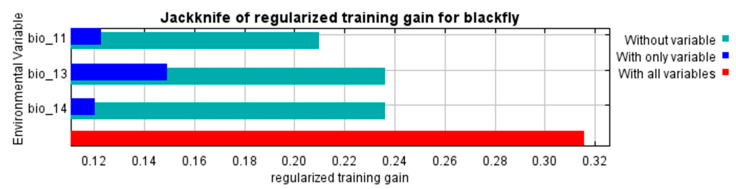
The relative importance of environmental variables based on the Jackknife test of regularized training gain in the CBF model.

The Jackknife test showed which bioclimatic variables had the greatest influence on CBF suitability. The following figure shows the results of the jackknife test according to variable importance. The bioclimatic variable with the highest gain, when used in isolation, is bio13, which appears to have the most helpful information. The bioclimatic variable that decreases the gain the most when it is omitted is bio11, which thus appears to have the most information that is not present in the other variables. The values shown are averages over replicate runs (Figure 2).

### 2.3. Response Curves

**Figure 3:**
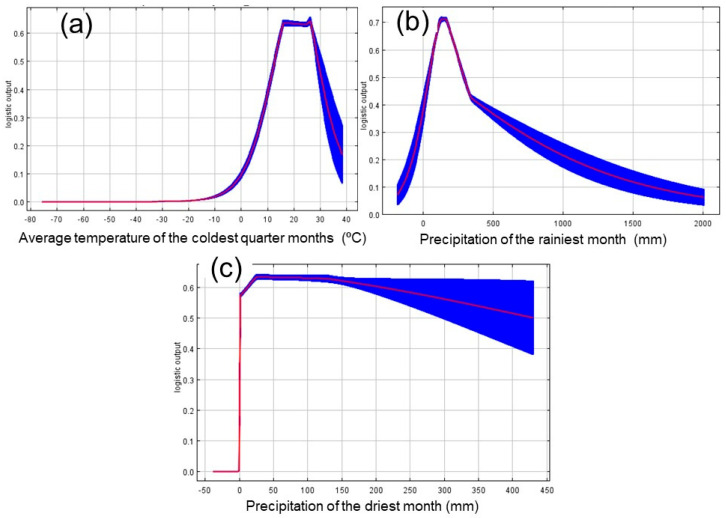
Response curves of the main predictors of CBF occurrence probability. The curves show the mean response over 10 replicate MaxEnt runs (red) and the mean ± one standard deviation (blue). (**a**) shows the response curve of the average temperature of coldest quarter months (°C), (**b**) shows the response curve of the precipitation of the rainiest moth (mm), and (**c**) shows the response curve of the precipitation of the driest month (mm).

The curves in Figure 3 show how the predicted probability of presence changes as each environmental variable is varied while keeping all other environmental variables at their average sample value.

When the average temperature of the coldest quarter months is between 15 and 25 °C, the probability of CBF presence is maximum, falling dramatically below or above this range (Figure 3a).

In regions where the precipitation of the rainiest month was around 200 mm, the CBF probability is at its maximum (above 0.7). However, when this precipitation rate exceeds 200 mm, the probability of pest presence falls quickly, reaching its minimum level (0.1) in regions with precipitation above 1500 mm (Figure 3b).

Regarding precipitation of the driest month, the probability of pest occurrence is zero in regions with no precipitation in the driest month. However, any precipitation that occurs in that month can increase the probability of occurrence of the pest, with its maximum (0.6) being reached in regions that have 50 to 150 mm of precipitation in the driest month. (Figure 3c).

### 2.4. Current Potential Distribution of CBF

**Figure 4:**
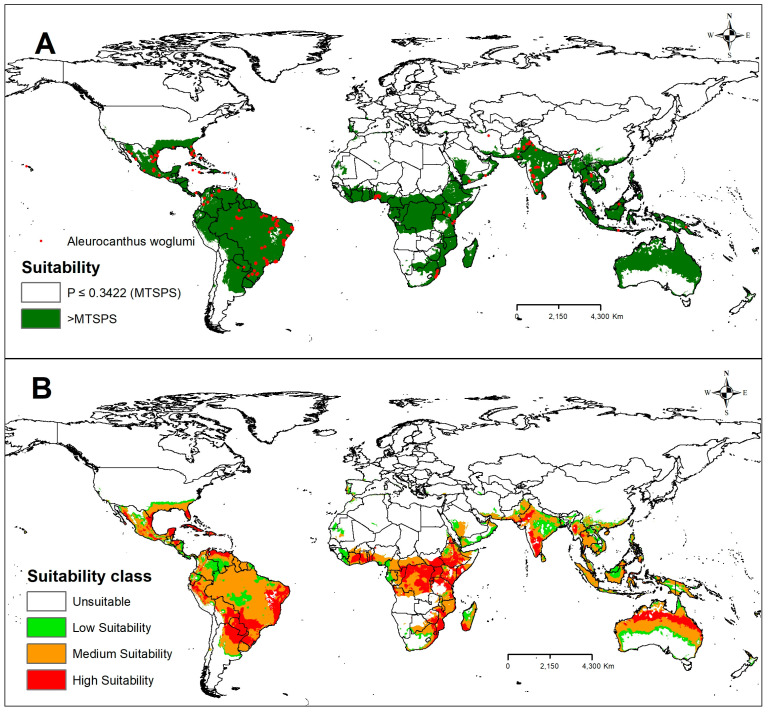
(**A**) Global known distribution and current areas suitable for CBF (P = probability) and (**B**) class of suitability under current climatic conditions. Unsuitable: P ≤ 0.3422, low suitability: 0.3422 < P ≤ 0.4, medium suitability: 0.4 < P ≤ 0.6, and high suitability: P > 0.6.

Currently, CBF is found in almost all continents favorable to its occurrence, except in the European continent and the polar regions (Figure 4A). It has been observed that most of the points of occurrence reported in this study are located in tropical and subtropical regions, with an emphasis on the regions with greater suitability of Ecoclimatic Index (MTSPS) favorable to the occurrence of CBF located in the southern part of North America, Central America, South America, south-central Africa, and South Asia (Figure 4A). In addition, most of these occurrence points are in areas identified as regions of medium (medium suitability) to high levels of climatic suitability (high suitability) for CBF (Figure 4B), giving high reliability to the model reported in this study. In addition, building models based on locations where the species already occurs is important for validating forecast models and adjusting variables with greater biological weight for the establishment and development of the species of interest.

The American continent had the largest area with climatic suitability for CBF. In North America, Mexico and the southern region of the United States were the areas that showed the greatest suitability for CBF (Figure 4B). In Central America, all regions were found to be suitable for CBF (Figure 4B). In South America, only Argentina, Chile, Bolivia, and Peru had areas considered unsuitable for the development of CBF (Figure 4B). On the African continent, areas with climate suitability for CBF included some countries from the west to the southeast of Africa and the island of Madagascar (Figure 4B). In Europe, all areas were considered unsuitable for the development of CBF (Figure 4B). On the Asian continent, the regions with the greatest suitability for CBF were Pakistan, India, South China, Thailand, Vietnam, Cambodia, and Indonesia (Figure 4B). In Oceania, all areas were suitable for CBF, except for the desert region of Australia and southern New Zealand (Figure 4B). In this region, coastal areas in the north of Australia were most suitable for CBF (Figure 4B).

### 2.5. Impact of Climate Change on Future Potential Distribution of CBF in 2030 and 2050 under the SSP1-2.6 Scenario

**Figure 5:**
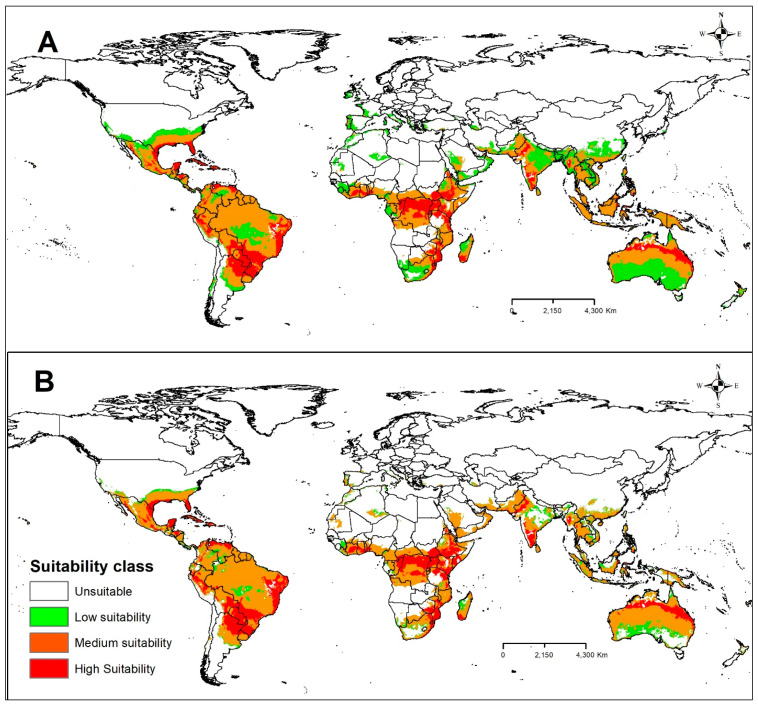
Class of suitability for CBF under scenario SSP1-2.6 in 2030 (**A**) and 2050 (**B**). Unsuitable: P ≤ MTSPS, low suitability: MTSPS < P ≤ 0.4, medium suitability: 0.4 < P ≤ 0.6, and high suitability: P > 0.6. P = probability.

The results of the MaxEnt model under future SSP1-2.6 climate change scenarios for 2030 and 2050 are presented in Figure 5A,B. According to the results, a small expansion of areas of low and medium climatic suitability for CBF is projected in North America for the years 2030 and 2050 in the south of the USA (Figure 5A,B). In South America, an increase in areas of medium and high climate suitability for CBF is projected for part of the north, west-central, and northeast regions of Brazil in the near future (Figure 5A,B). Small reductions of medium suitability areas are only observed in northern Brazil, northern Colombia, and southern Ecuador. In Africa, a slight increase in the areas of low and medium climatic suitability is projected in some North African countries and South Africa between 2030 and 2050 (Figure 5A,B), with a slight reduction of areas of high suitability in the Southern African region, especially in the Democratic Republic of Congo in 2050 (Figure 5B). In Europe, potential areas of low suitability for CBF are projected to emerge in the southernmost region of the continent by 2030 (Figure 5A). In this scenario, countries such as Portugal, Spain, France, Ireland, Italy, Greece, and Turkey would have areas susceptible to this insect pest. However, only Portugal stands out with the largest suitability area for CBF in the 2050 scenario (Figure 5B). In Asia, an increase in the range of low and medium climate suitability is projected in southern China for the year 2030, and a slight expansion of the range of medium suitability is projected for 2050 (Figure 5B). The most significant change was observed for the 2050 projection, with a reduction in the significance of the CBF suitability area in central India (Figure 5B). In Oceania, future climate simulations project an expansion of low- and medium-suitability areas for CBF in central and southern Australia in the 2030 scenario (Figure 5A). However, in the 2050 scenario, there is a slight increase in the medium climate suitability range in the central region and the unfavorable areas of southern Australia. In addition, the model also projected a progressive contraction of the low-suitability regions, especially in the south of the country (Figure 5B).

### 2.6. Impact of Climate Change on Future Potential Distribution of CBF in 2030 and 2050 under the SSP5-8.5 Scenario

**Figure 6:**
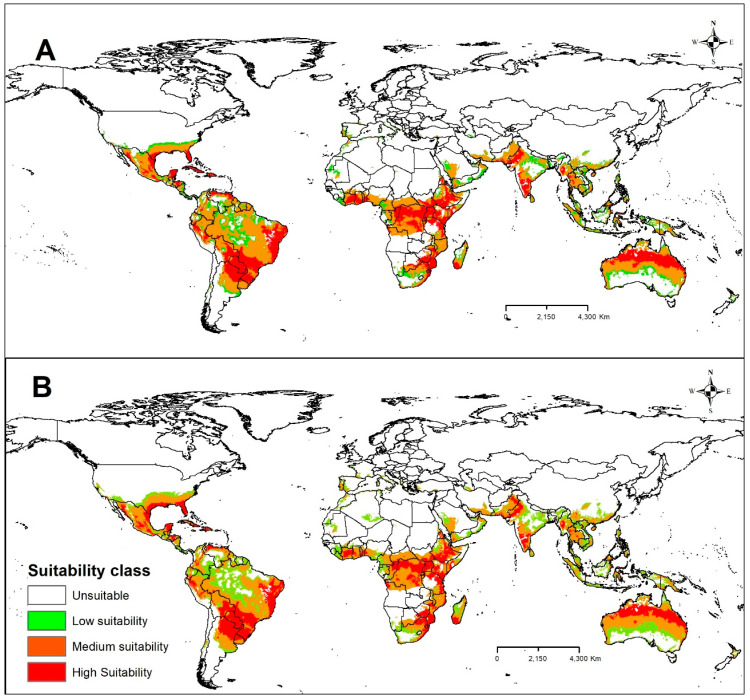
Class of suitability for CBF under scenario SSP5-8.5 in 2030 (**A**) and 2050 (**B**). Unsuitable: P ≤ MTSPS, low suitability: MTSPS < P ≤ 0.4, medium suitability: 0.4 < P ≤ 0.6, and high suitability: P > 0.6. P = probability.

The results of the MaxEnt model under future SSP1-2.6 climate change scenarios for 2030 and 2050 are presented in Figure 6A,B. In this model, in North America, a small expansion of areas of low, medium, and high climate suitability for CBF is projected for the years 2030 and 2050, especially in the southern region of the USA (Figure 6A,B). In South America, a retraction of medium and high climate suitability areas for CBF is projected for part of the northern regions (especially in the Amazon region) and west-central Brazil, northern Colombia, and the central and southern regions of Venezuela in the future (Figure 6A,B). On the other hand, a significant increase in areas of low suitability is projected for the northern and mid-west regions of Brazil. In Africa, a slight expansion of medium and high climatic suitability areas is projected for the northeast and north of South Africa, especially in 2050 (Figure 6A,B). A slight reduction of areas of high suitability is projected for the Southern African region, especially in the Democratic Republic of Congo, in 2050 (Figure 6B). Only Portugal stands out in Europe with the greatest suitability area for CBF, especially in the 2050 scenario (Figure 6B). In Asia, a slight expansion of the low and medium climate suitability range is projected for southern China for 2030 and 2050 (Figure 6A,B). However, low and medium CBF suitability areas are predicted to decline significantly in the western, central, and eastern parts of India (Figure 6B). In Oceania, future climate simulations project a slight expansion of the range of high suitability for CBF in northern Queensland, Northern Territory, and Western Australia in the 2030 scenario (Figure 6A). However, in the 2050 scenario, there is a slight increase in the range of low and medium climate suitability and a reduction in the range of high climate suitability (Figure 6B).

### 2.7. Global Orange Production

**Figure 7:**
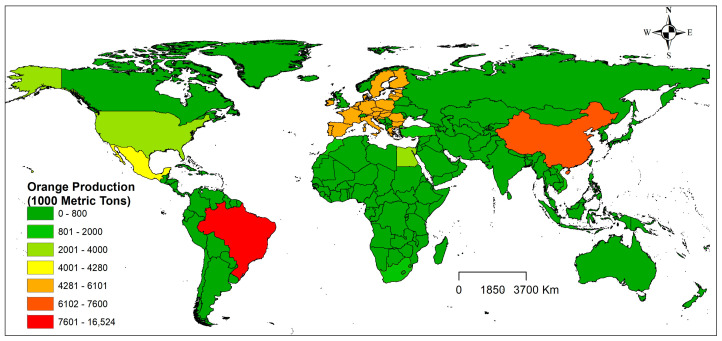
Global representation of the countries with the highest orange production in the world in the current scenario.

Global orange production for 2021/2022 is estimated at 49.0 million. In this scenario, Brazil stands out as the largest producer of oranges, with a harvest of 16.9 million tons. China is the world’s second-largest producer, with a crop of 7.6 million tons, according to [25]. (Figure 7). The European Union is the third largest producer in the world, with a harvest of 6.1 million tons. USA, Egypt, Turkey, and Morocco occupy the fourth, fifth, sixth, and seventh positions in the ranking of the largest orange-producing countries, with an estimated production of 3.5, 3.0, 1.8, and 1.2 million tons, respectively.

## 3. Materials and Methods

### 3.1. Occurrence Datasets

**Figure 8:**
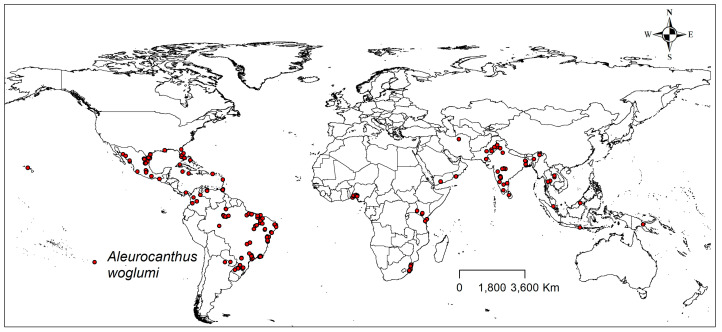
Current global distribution of CBF.

We extensively searched the scientific literature and databases to obtain worldwide occurrence records of CBF (Figure 8). For scientific literature searches, we looked for articles and scientific reports in online databases. For database searches, we used the Global Biodiversity Information Facility online database.

To improve occurrence data, we removed duplicate occurrences, and records related to occurrences in the sea were adjusted or removed using Google Earth PRO to edit the latitude and longitude [26,27].

To reduce the possible bias in the occurrence point, we applied spatial filtering using spThin, an R package [28]. After this, 266 remaining occurrences were retained for the model, ensuring that each cell had only a single occurrence record [28,29].

### 3.2. Bioclimatic Variables Data

We chose twenty bioclimatic variables from the WorldClim version 2.1 as initial predictors for the CBF model (Table 4). The layers had about 5 km spatial resolution, which was suitable for supporting global scale modeling. WorldClim projects current conditions based on observations from different weather stations between 1970 and 2000.

The choice of final bioclimatic variables is crucial to the accuracy of the model. Therefore, we used a method to select the best bioclimatic variables for our data.

The approach to select the best variables for the final model from the 20 initial variables was as follows: (1) We established an initial model to calculate the contribution of the 20 variables to the model (Table 1). (2) We used ArcGIS to extract the attribute values for 20 variables at each of the 271 presence records and calculated the Pearson correlation coefficients between any two variables. (3) If a correlation coefficient was greater than 0.7, the most relevant variable was retained according to its percent contribution in the initial model, and the other variable was excluded. (4) The remaining variables were sorted according to their percent contributions, and only those with more than 1% were retained in the final model. Through the above procedure, we completed the selection of bioclimatic variables for the final model.

### 3.3. Future Projections for Aleurocanthus woglumi

We projected two future periods (2030 and 2050) under two different Intergovernmental Panel on Climate Change (IPCC) scenarios (SSP1-2.6 and SSP5-8.5) from the sixth assessment report (AR6). These data were the mean of the monthly values over 20-year periods (2021–2040 and 2041–2060). The SSP1-2.6 represents a more positive scenario in which protection measures are being taken. In contrast, the SSP5-8.5 scenario represents a more pessimistic scenario described in the AR6. The MIROC-6 (Model for Interdisciplinary Research on Climate) was the Global Climate Model (GCM) employed because it is a newly developed climate model, with updates to its physical parameterizations in all sub-modules.

### 3.4. MaxEnt Development and Validation

First, we created a MaxEnt initial model with CBF occurrence and the 20 bioclimatic variables. In this initial model, we selected ‘Do jackknife to measure variable importance’ to calculate the contribution of each bioclimatic variable. Considering that we did not control the occurrence sampling process, a sampling bias surface was also developed using the kernel density estimate available in the SDMToolbox 2.6. Finally, to choose the bioclimatic variables for the final model, we assessed their percentage contributions and their Pearson coefficient, as described in Section 2.2. The final CBF model was based on adjusting the default MaxEnt settings for certain combinations of resource types and the regularization multiplier (RM) [20,30,31]. We combined linear (L), quadratic (Q), product (P), threshold (T), and hinge (H) feature sets using automatic feature selection plus RM = 1 to control the number of parameters and, therefore, the complexity of the model for the species [32]. In addition, concerning the modeling accuracy, we evaluated the AUC (area under the receiver operating characteristic [ROC] curve).

A 10-repeat cross-validation was run in MaxEnt to calculate the AUC. AUC values of 0.5 indicate that predictions are random, values < 0.5 are even worse than random; values between 0.5 and 0.7 indicate poor performance, values between 0.7 and 0.9 indicate reasonable performance, and values > 0.9 indicate elevated performance. The variable’s contributions were estimated by the Jackknife test.

MaxEnt generates a probability suitability index (P) for the species ranging from 0 (for unsuitable) to 1 (for optimum suitability). The maximum test sensitivity plus specificity threshold (MTSPS) was chosen to divide the model outputs into suitable and unsuitable areas for CBF because MTSPS is considered simple, effective, and as good as more complex methods. The model outputs were also further regrouped into four categories: Unsuitable: P ≤ MTSPS, low suitability: MTSPS < P ≤ 0.4, medium suitability: 0.4 < P ≤ 0.6, and high suitability: P > 0.6.

Response curves were also developed in MaxEnt, and we only chose those that provided powerful biological logic, indicating that the species suitability does not show many fluctuations as the bioclimatic variables increase.

## 4. Discussion

The intensification of globalization and climate change in recent centuries has caused significant threats to the dynamics of ecosystems and favored the accidental or intentional introduction of invasive alien species such as CBF in various regions of the world. To understand this process and protect these regions, we developed this study to assess the potential suitable area for the invasive CBF pest under current and future climate change scenarios on a global scale by MaxEnt to avoid commission errors in predicting species distribution [33]. According to the pROC results, the models reported in this study are robust and of excellent performance, presenting values greater than 93 and 95.3% of AUC (Figure 1) and 100% of the records of occurrences in the validation area (Figure 4A,B). The current distribution of CBF is much smaller than the habitat predicted as suitable by MaxEnt. Under scenarios of future climate changes in the coming decades (2030 to 2050), our models indicate a variation in the size of areas favorable to this insect pest. In this context, global and quarantine economic measures are urgent to mitigate the expansion of CBF in areas favorable to its occurrence, especially in Europe and the Mediterranean Basin, where this species is unknown.

Climatic factors such as temperature and precipitation play a crucial role in the potential geographic distribution of CBF, as reported in our MaxEnt model results. We identified that the environmental variables bio11 (36.1%), bio13 (32.4%), and bio14 (31.6%) were the most important variables for the current determination of the potential distribution of this insect pest (Table 3). The response curve showed that the probability of CBF was maximum when the average temperature of the coldest quarter (bio11) is between 15 and 25 °C, falling drastically below or above this range (Figure 3a). These values are in line with the optimum temperature for CBF development, which ranges from 26 to 32 °C [34], but this species can withstand a minimum temperature of up to 14 °C and a maximum of 43 °C [12,35]. However, negative effects are expected when insects are subjected to temperatures close to the minimum and maximum limits they can withstand [36]. In addition, the potential distribute on of CBF in the present study is suitable for the ideal temperature range for the good development and production of citrus plants, which is between 25 and 30 °C during the day and between 10 and 15 °C at night [37]. The orange tree and other citrus trees prefer climates with temperatures between 23 and 32 °C and high relative humidity (75 to 80%). Above 40 °C and below 13 °C, the photosynthesis rate decreases, leading to productivity losses [38].

On the other hand, the probability was maximum in regions with precipitation of up to 200 mm in the wettest month (bio13) (Figure 3b) and from 50 to 150 mm of precipitation in the driest month (bio14) (Figure 3c). In these cases, the response curves show that the probability of the presence of CBF was very low when this precipitation rate exceeded 200 mm in the wettest month or zero (0) in regions without precipitation in the driest month. Several studies have reported the importance of rainfall as an agent capable of dislodging and killing adult insect pests [39,40,41], negatively affecting the CBF population dynamics in the field [42]. The ideal rainfall regime for citrus orchards ranges from 1000 to 1800 mm, indicating that the precipitation regime influences the establishment of the citrus culture and the insect pest in a similar way. However, in places where there is a water deficit, this problem can be circumvented by using irrigation, mainly in areas in the northeast of Brazil where the rainfall regime is below 700 mm [43]. However, this did not interfere with the quality of the global area model with suitability for CBF since high-altitude regions, where the Andes Mountains (especially Chile and Argentina) and the Himalayas are located, were considered unsuitable for this species.

In this study, the potential distribution model of CBF was suitable for predicting the occurrence of this insect pest in all continents where there is a record and favorable conditions for this species (Figure 4A,B), except in the European continent and at the poles. The MaxEnt model was implemented based on information from occurrence points and corresponding environmental variables based on the maximum entropy theory [44]. According to [45], the model’s accuracy based on MaxEnt is mainly affected by occurrence data and environmental data. To avoid possible errors, the points of occurrence reported in this study were collected in locations located in tropical and subtropical regions with very different climatic characteristics, with an emphasis on the areas with the greatest suitability of the Ecoclimatic Index (MTSPS) favorable to the occurrence of CBF located in the southern part of North America, Central America, South America, south-central Africa, and the southern part of Asia (Figure 4A,B). In addition, building models based on locations where the species already occurs is important for validating forecast models and adjusting variables with greater biological weight for the establishment and development of the species of interest. As reported in other studies [46,47], climate variables were also considered as the main factors influencing the distribution of CBF on a large global scale based on the current model and data. In addition, we found that climatic factors favored both the occurrence of the insect pest and the host species. Other factors such as the study area, dispersal capacity, agricultural management of the area, size, and architecture of citrus plants, availability and nutritional quality of the host, nutrition in the immature stages, light intensity, relative humidity [3,48,49], and natural enemies (for example predators and parasitoids) [10] can also influence the results of the CBF potential distribution model.

The current study showed that, under present conditions, the global adequacy of CBF is concentrated in parts of the tropical and subtropical regions of America (North, Central, and South), Asia, the Pacific, and Africa (central and southern) (Figure 4A); there are no records of occurrence in Europe and the polar regions. The current distribution findings in the present study do not differ from the findings of [24], who used CLIMEX, indicating that areas of the Sahara Desert, central Australia, New Zealand, Korea, most of Europe, China, the USA, Canada, and Russia are among the countries or regions with unfavorable conditions for the occurrence of CBF. In our model, the main restrictions occurred due to the environmental variable bio11, which identified the limitation of the occurrence of CBF in regions with temperatures below 14 °C and above 43 °C [12,35], especially in the cold areas such as northern Europe, China, the USA, Canada, and Russia. Places with a high-temperature range, such as the Sahara Desert, can have a temperature variation of 50 °C (day) to −10 °C (night), making it impossible for this species to occur in the region. On the other hand, the environmental variable bio13 indicated a limitation of the occurrence of CBF in regions with a monsoon climate such as in the vicinity of the Himalayas; precipitation in these areas can reach 3000 mm in 4 months. In the southern part of Asia, this volume can reach 5000 mm, a volume well above 200 mm in the wettest month supported by the species. Interestingly, the Amazon region presents ideal development conditions, i.e., temperature between 28 and 32 °C and relative humidity between 70 and 80% [40,50], allowing the occurrence of five to six annual generations, average production of 26 to 58 per posture (interval of 7 to 48 days), and average production of 1845 adults per couple over a year [51]. However, in the region of the mouth of the Amazon River and the northwest sector of the Amazon, we identified a limited suitability for CBF, due to the annual precipitation being greater than 3000 mm in this region [52]. The environmental variable bio14 indicated a limitation of the occurrence of CBF in places with a semi-arid climate such as the northeast region of Brazil, where annual values below 1000 mm are observed [52,53]. However, this potential distribution area may change with the adoption of irrigation systems, as reported by [24].

In the future climate change scenarios SSP1-2.6 and SSP5-8.5, areas highly suitable for CBF were mainly found to occur in North America, Africa, Europe, Asia, and Oceania. Our study demonstrated that areas of global bioclimatic suitability for CBF are expected to increase under both low SSP1-2.6 and high SSP5-8.5 emission scenarios, mainly in countries with subtropical climates. A priori, the increase in average global temperature of 2 °C predicted in the SSP1-2.6 scenario between 2030 and 2050 favors the expansion of CBF mainly in areas located in the south of the USA, southern Europe, North Africa, southern China, and part of Australia. On the other hand, the more pessimistic scenario of SSP5-8.5, mainly in 2050, caused a great retraction in the areas suitable for CBF in the tropical region, particularly in countries such as Brazil, Colombia, Venezuela, and India. The projections for an increase in temperature estimated until 2050 [22] for these areas probably made the survival and maintenance of this species unfeasible. The predictions under the SSP5-8.5 high emission scenario (Figure 6A,B) in the current study differ from the findings of [24]. They predicted a potential increase in areas suitable for CBF on all continents in a more pessimistic RCP 8.5 scenario by 2050, except in Africa. This possible expansion attributed to the relaxation of cold stresses was limited and offset by decreased range due to lethal heat or dry stress. These results are more similar to our prediction model in the SSP1-2.6 scenario (Figure 5A,B), where the expansion of areas favorable to CBF in the tropical and subtropical regions was evident. Changes in CBF suitability may be because they used CLIMEX models, which differ from the MaxEnt models used in the present study. The multi-model ensemble predicted the highest risk of CBF invasion and spread in 2030 and 2050 in the SSP1-2.6 scenario (Figure 5A,B). This study also demonstrated that suitable habitats for CBF are increasing dramatically around the world in scenarios of future climate change, which may be due to the increase in temperature and annual precipitation that are favoring the emergence of new areas more suitable for the survival of this insect pest.

Brazil has the most significant area with climatic suitability for the CBF, with approximately 90% of the country’s area classified as medium to high suitability for this insect pest (Figure 4B). However, Brazil is the world leader in citrus production, producing over 1.1 million tons (Figure 7). In the northeast, the area of high suitability starts in Ceará and cuts through all the states in the northeast, reaching Rio de Janeiro, in the southeast region. In the southeast, the area of high suitability starts in São Paulo, and passes through Triângulo Mineiro, Paraná (southern region), until it reaches Mato Grosso do Sul, in the mid-west region. In the south, the area of high suitability for the CBF is located mainly in Rio Grande do Sul (Figure 4B). Notably, the areas of high suitability for the CBF identified in the models coincide with the largest citrus production centers in the country. In this context, the central Brazilian citrus belt located in the southeast region concentrates 84% of national production, with São Paulo being the largest producer (78%), followed by Minas Gerais (6%) [54]. The list of the six largest citrus producers in the country also includes the states of Paraná (4.1%), Bahia (3.5%), Sergipe (2.1%) and Rio Grande do Sul (2.0%) [54]. Although these predicted habitats for CBF in Brazil by the MaxEnt model in the current study are excellent, we recognize that the models have some limitations that may not explain the spatial bias of occurrence records [55]. Factors such as host plants, predators, parasitoids, and management practices are not generally considered in ecological niche models, but the interspecific interactions of these factors can significantly affect species distribution [56]. However, based on the models, there is a need for management and monitoring strategies to avoid the increase in losses caused by CBF in areas where this species is already registered and a reinforcement of phytosanitary defense agencies to create policies aimed at mitigating the dispersion of this insect pest, mainly in the citrus belt regions or in regions, where there is no record of this insect pest.

## 5. Conclusions

The model performed in MaxEnt to predict the global suitability of CBF under current conditions and future SSP1-2.6 and SSP5-8.5 climate change scenarios indicated greater adequacy of habitat for CBF in Brazil, China, the European Union, the USA, Egypt, Turkey, and Morocco, which are the world’s largest orange producers. Therefore, consolidated efforts must be placed on controlling the distribution and mitigating the proliferation of CBF in these regions, both in the present and in future scenarios. In continents with high habitat suitability, where CBF is an important quarantine species, governments should prioritize the adoption of strict quarantine measures such as intensifying actions aimed at inspection, increasing phytosanitary barriers, and combating possible illegal entry routes of host plant material on highways, ports, and airports, to prevent the invasion of this insect pest in the country. On the other hand, in countries where the occurrence of CBF is restricted to some regions, actions should be promoted, aimed at promoting greater phytosanitary rigor in the production and sale of citrus seedlings or ornamental host plants for this insect, eliminating possible outbreaks of infestation of populations of this insect pest in alternative hosts in commercial and residential crops and promoting management actions and awareness of producers about the importance of handling and controlling this insect pest. Adopting these measures will not end the problems caused by CBF in citriculture. However, these actions are important to mitigate this pest agent’s reproduction, maintenance, and dissemination in nurseries and commercial plantations, mainly in the citrus belt regions where the economic impact caused by this pest is significant.

## Figures and Tables

**Table 1:** Pairwise Pearson’s correlation coefficients of environmental variables from the initial model. The variables selected for the final model are highlighted in bold.

Bio	1	2	3	4	5	6	7	8	9	10	11	12	13	14	15	16	17	18	19	20
1	1.00																			
2	0.03	1.00																		
3	0.72	−0.56	1.00																	
**4**	−0.71	0.60	−0.98	1.00																
5	0.05	0.99	−0.60	0.62	1.00															
**6**	0.63	−0.73	0.95	−0.98	−0.73	1.00														
7	−0.36	0.91	−0.85	0.88	0.91	−0.95	1.00													
**8**	−0.56	−0.12	−0.47	0.53	−0.07	−0.38	0.20	1.00												
9	0.88	−0.19	0.86	−0.88	−0.22	0.79	−0.58	−0.81	1.00											
**10**	−0.14	0.92	−0.77	0.79	0.95	−0.84	0.96	0.20	−0.46	1.00										
11	0.88	−0.38	0.95	−0.96	−0.39	0.91	−0.73	−0.59	0.95	−0.59	1.00									
12	0.24	−0.85	0.77	−0.75	−0.87	0.82	−0.91	0.12	0.36	−0.87	0.60	1.00								
13	0.39	−0.63	0.80	−0.76	−0.67	0.78	−0.79	0.02	0.46	−0.74	0.68	0.94	1.00							
**14**	0.01	−0.65	0.27	−0.22	−0.58	0.39	−0.51	0.68	−0.16	−0.39	0.13	0.68	0.53	1.00						
**15**	0.29	0.89	−0.18	0.23	0.83	−0.43	0.65	−0.48	0.20	0.64	−0.02	−0.67	−0.41	−0.77	1.00					
16	0.43	−0.69	0.86	−0.81	−0.73	0.83	−0.84	0.00	0.50	−0.79	0.72	0.96	0.98	0.59	−0.44	1.00				
17	0.02	−0.69	0.32	−0.28	−0.64	0.44	−0.56	0.64	−0.12	−0.46	0.17	0.72	0.57	1.00	−0.80	0.63	1.00			
**18**	−0.66	−0.60	−0.28	0.26	−0.54	−0.07	−0.21	0.68	−0.61	−0.27	−0.46	0.25	−0.05	0.63	−0.85	0.00	0.63	1.00		
**19**	0.64	−0.61	0.96	−0.94	−0.64	0.93	−0.86	−0.28	0.74	−0.77	0.89	0.88	0.93	0.40	−0.28	0.95	0.44	−0.22	1.00	
20	0.26	0.92	−0.40	0.38	0.93	−0.51	0.75	−0.39	0.07	0.81	−0.14	−0.83	−0.62	−0.74	0.87	−0.67	−0.77	−0.70	−0.49	1.00

Bio1: annual average temperature, bio2: average variation in daytime temperature, bio3: isothermality, bio4: seasonality of temperature, bio5: highest temperature of the hottest month, bio6: lowest temperature of the coldest month, bio7: annual temperature variation, bio8: average temperature of the rainiest quarter months, bio9: average temperature of the driest quarter months, bio10: average temperature of the hottest quarter months, bio11: average temperature of the coldest quarter months, bio12: annual precipitation, bio13: precipitation of the rainiest month, bio14: precipitation of the driest month, bio15: precipitation seasonality, bio16: precipitation of the rainiest quarter months, bio17: precipitation of the driest quarter months, bio18: precipitation of the hottest quarter months, bio19: precipitation of the coldest quarter months, and bio20: elevation.

**Table 2:** Percent contribution of the environmental variables used in the initial Maxent model.

Code	Environmental Variable	Percent Contribution
bio11	Average temperature of the coldest quarter months	36.7
bio13	Precipitation of the rainiest month	14.7
bio3	Isothermality	13.2
bio14	Precipitation of the driest month	10.2
bio4	Seasonality of temperature	5.2
bio20	Elevation	4
bio19	Precipitation of the coldest quarter months	2.5
bio17	Precipitation of the driest quarter months	2.4
bio16	Precipitation of the rainiest quarter months	2.2
bio1	Annual average temperature	1.8
bio6	Lowest temperature of the coldest month	1.4
bio2	Average variation of daytime temperature	1.3
bio12	Annual precipitation	1.2
bio7	Annual temperature variation	1.2
bio18	Precipitation of the hottest quarter months	0.6
bio10	Average temperature of the hottest quarter months	0.5
bio15	Precipitation seasonality	0.3
bio5	Highest temperature of the hottest month	0.2
bio9	Average temperature of the driest quarter months	0.2
bio8	Average temperature of the rainiest quarter months	0.1

**Table 3:** Percent contribution of the environmental variables used in the final MaxEnt model.

Code	Environmental Variable	Percent Contribution
bio11	Average temperature of the coldest quarter months	36.1
bio13	Precipitation of the rainiest month	32.4
bio14	Precipitation of the driest month	31.6

**Table 4:** Variables initially used in the CBF model.

Code	Environmental Variable	Unit
bio1	Annual average temperature	°C
bio2	Average variation in daytime temperature	°C
bio3	Isothermality	°C
bio4	Seasonality of temperature	°C
bio5	Highest temperature of the hottest month	°C
bio6	Lowest temperature of the coldest month	°C
bio7	Annual temperature variation	°C
bio8	Average temperature of the rainiest quarter months	°C
bio9	Average temperature of the driest quarter months	°C
bio10	Average temperature of the hottest quarter months	°C
bio11	Average temperature of the coldest quarter months	°C
bio12	Annual precipitation	mm
bio13	Precipitation of the rainiest month	mm
bio14	Precipitation of the driest month	mm
bio15	Precipitation seasonality	mm
bio16	Precipitation of the rainiest quarter months	mm
bi017	Precipitation of the driest quarter months	mm
bio18	Precipitation of the hottest quarter months	mm
bio19	Precipitation of the coldest quarter months	mm
bio20	Elevation	m

## Data Availability

Data are contained within the article.

## References

[1] Rapisarda C., Massimino Cocuzza G.E., Marano G., Conti F. (2016). Emergenze Fitosanitarie: Aspetti Entomologici.

[2] EPPO Global Database—European Union Funding. https://gd.eppo.int/taxon/ALECWO/distribution.

[3] Vieira D.L., Batista J., De Oliveira R., Malaquias J.B., De Souza G.M. (2017). *Aleurocanthus woglumi* (Hemiptera: Aleyrodidae) in Citrus: Opportunities and Challenges to Implement a Sustainable Management. Citrus Pathology.

[4] Nguyen R., Hamon A.B., Fasulo T.R. Citrus Blackfly, *Aleurocanthus woglumi* Ashby (Insecta: Hemiptera: Aleyrodidae). https://edis.ifas.ufl.edu/pdf%5CIN%5CIN19900.pdf.

[5] Smith H.D., Maltby H.L., Jiménez E.J. (1964). Biological Control of the Citrus Blackfly in Mexico.

[6] Bedford E., Thomas E. (1965). Biological Control of the Citrus Blackfly, *Aleurocanthus woglumi* (Ashby) (Homoptera: Aleyrodidae) in South Africa. J. Entomol. Soc. S. Afr..

[7] Maia W.J.M.S., Souza J.C., Marques L.C., Silva L.M.S., Benaduce R.V., Gentil R.M. Infestação em citros por *Aleurocanthus woglumi* (Ashby) e perspectivas de Controle biológico aplicado no Pará. Proceedings of the Anais do 9° Simpósio de Controle Biológico.

[8] Barbosa F., Santana M.d.R., da Silva C., Paranhos B. (2004). Aleurocanthus woglumi (Hemíptera: Aleyrodidae): Uma Ameaça À Fruticultura Do Vale Do São Francisco.

[9] De Moraes B.C., De Souza E.B., Ribeiro J.B.M., Ferreira D.B.D.S., Maia W.J.d.M. (2014). Impactos das mudanças climáticas na ecoclimatologia de *Aleurocanthus woglumi* Ashby, 1903 (Hemiptera: Aleyrodidae) no estado do Pará. Rev. Bras. Meteorol..

[10] Aruna J., Jagginavar S., Karabhantanal S., Huilgol S.N. (2017). Seasonal Incidence of Citrus Blackfly, *Aleurocanthus woglumi* Ashby and Its Natural Enemies on Acid Lime. J. Exp. Zool. India.

[11] Lima B.G., Farias P.R.S., Ramos E., Soares M.L., Sales T.D.M., Silva A.G.D. (2017). Economic Injury Level of Citrus Black-Fly in Commercial ‘Pera-Rio’orange Area. Rev. Bras. Frutic..

[12] Health E.P.o.P., Bragard C., Dehnen-Schmutz K., Di Serio F., Gonthier P., Jacques M.A., Jaques Miret J.A., Justesen A.F., Magnusson C.S., Milonas P. (2018). Pest Categorisation of *Aleurocanthus* spp.. EFSA J..

[13] Mingoti R., Pessoa M., Marinho-Prado J., Siqueira C., Ramos G., Jacomo B., Catarina De Araújo Siqueira B.C., Giovanna Galhardo Ramos B.C., Bárbara De Oliveira Jacomo B.C. (2021). Zoneamentos Mensais de Áreas Favoráveis a Aleurocanthus Woglumi No Brasil.

[14] Anderson R.P., Martínez-Meyer E., Nakamura M., Araújo M.B., Peterson A.T., Soberón J., Pearson R.G. (2011). Ecological Niches and Geographic Distributions (Mpb-49).

[15] Kumar S., Neven L.G., Yee W.L. (2014). Evaluating Correlative and Mechanistic Niche Models for Assessing the Risk of Pest Establishment. Ecosphere.

[16] Santana P.A., Kumar L., Da Silva R.S., Pereira J.L., Picanço M.C. (2019). Assessing the Impact of Climate Change on the Worldwide Distribution of *Dalbulus maidis* (Delong) Using Maxent. Pest Manag. Sci..

[17] Yan X.-R., Wang Z.-Y., Feng S.-Q., Zhao Z.-H., Li Z.-H. (2022). Impact of Temperature Change on the Fall Armyworm, *Spodoptera frugiperda* under Global Climate Change. Insects.

[18] Ramasamy M., Das B., Ramesh R. (2022). Predicting Climate Change Impacts on Potential Worldwide Distribution of Fall Armyworm Based on Cmip6 Projections. J. Pest Sci..

[19] Galdino T.V.d.S., Kumar S., Oliveira L.S., Alfenas A.C., Neven L.G., Al-Sadi A.M., Picanco M.C. (2016). Mapping Global Potential Risk of Mango Sudden Decline Disease Caused by *Ceratocystis fimbriata*. PLoS ONE.

[20] Aidoo O.F., da Silva R.S., Santana Junior P.A., Souza P.G.C., Kyerematen R., Owusu-Bremang F., Yankey N., Borgemeister C. (2022). Model-Based Prediction of the Potential Geographical Distribution of the Invasive Coconut Mite, *Aceria guerreronis Keifer* (Acari: Eriophyidae) Based on Maxent. Agric. For. Entomol..

[21] Ramos R.S., Kumar L., Shabani F., Picanço M.C. (2018). Mapping Global Risk Levels of Bemisia Tabaci in Areas of Suitability for Open Field Tomato Cultivation under Current and Future Climates. PLoS ONE.

[22] IPCC (2019). Climate Change and Land: An IPCC Special Report on Climate Change, Desertification, Land Degradation, Sustainable Land Management, Food Security, and Greenhouse Gas Fluxes in Terrestrial Ecosystems.

[23] Gidden M.J., Riahi K., Smith S.J., Fujimori S., Luderer G., Kriegler E., van Vuuren D.P., van den Berg M., Feng L., Klein D. (2019). Global Emissions Pathways under Different Socioeconomic Scenarios for Use in Cmip6: A Dataset of Harmonized Emissions Trajectories through the End of the Century. Geosci. Model Dev..

[24] Akrivou A., Georgopoulou I., Papachristos D.P., Milonas P.G., Kriticos D.J. (2021). Potential Global Distribution of *Aleurocanthus woglumi* Considering Climate Change and Irrigation. PLoS ONE.

[25] USDA (2022). Citrus: World Markets and Trade, Citrus World Mark. Trade. https://downloads.usda.library.cornell.edu/usda-esmis/files/w66343603/bv73d549r/1v53m4335/citrus.pdf.

[26] Zhang H., Song J., Zhao H., Li M., Han W. (2021). Predicting the Distribution of the Invasive Species *Leptocybe invasa*: Combining Maxent and Geodetector Models. Insects.

[27] Zhu Y., Wei W., Li H., Wang B., Yang X., Liu Y. (2018). Modelling the Potential Distribution and Shifts of Three Varieties of *Stipa tianschanica* in the Eastern Eurasian Steppe under Multiple Climate Change Scenarios. Glob. Ecol. Conserv..

[28] Aiello-Lammens M.E., Boria R.A., Radosavljevic A., Vilela B., Anderson R.P. (2015). spThin: An R Package for Spatial Thinning of Species Occurrence Records for Use in Ecological Niche Models. Ecography.

[29] Ramos R.S., Kumar L., Shabani F., da Silva R.S., de Araújo T.A., Picanço M.C. (2019). Climate Model for Seasonal Variation in *Bemisia tabaci* Using Climex in Tomato Crops. Int. J. Biometeorol..

[30] Jarnevich C.S., Stohlgren T.J., Kumar S., Morisette J.T., Holcombe T.R. (2015). Caveats for Correlative Species Distribution Modeling. Ecol. Inform..

[31] Merow C., Smith M.J., Silander J.A. (2013). A Practical Guide to Maxent for Modeling Species’ Distributions: What It Does, and Why Inputs and Settings Matter. Ecography.

[32] Elith J., Phillips S.J., Hastie T., Dudík M., Chee Y.E., Yates C.J. (2011). A Statistical Explanation of Maxent for Ecologists. Divers. Distrib..

[33] Townsend Peterson A., Papeş M., Eaton M. (2007). Transferability and Model Evaluation in Ecological Niche Modeling: A Comparison of Garp and Maxent. Ecography.

[34] Nguyen R. (2008). *Aleurocanthus* *woglumi*. Invasive Species Compendium.

[35] Dowell R.V., Fitzpatrick G.E. (1978). Effects of Temperature on the Growth and Survivorship of the Citrus Blackfly (Homoptera: Aleyrodidae) 1. Can. Entomol..

[36] Haddad M.L., Parra J.R.P., Moraes R.C.B. (1999). Métodos para Estimar os Limites Térmicos Inferior e Superior de Desenvolvimento de Insetos.

[37] Rodriguez O., Castro P.R.C., Ferreira S.O., Yamada T. (1987). Ecofisiologia dos Citros.

[38] Mattos Junior D.d., De Negri J., Figueiredo J.d., Pompeu Junior J. (2005). Citros: Principais Informações E Recomendações de Cultivo. Bol. Técnico.

[39] Flanders S.E., Herbert D. (1969). Smith’s Observations on Citrus Blackfly Parasites in India and Mexico and the Correlated Circumstances. Can. Entomol..

[40] Da Siva A.G., Farias P.R.S., Junior A.L.B., Souza B.H.S. (2011). Mosca-Negra-Dos-Citros: Características Gerais, Bioecologia E Métodos De Controle Dessa Importante Praga Quarentenária Da Citricultura Brasileira. EntomoBrasilis.

[41] Rodrigues-Silva N., de Oliveira Campos S., de Sá Farias E., De Souza T.C., Martins J.C., Picanço M.C. (2017). Relative Importance of Natural Enemies and Abiotic Factors as Sources of Regulation of Mealybugs (Hemiptera: Pseudococcidae) in Brazilian Coffee Plantations. Ann. Appl. Biol..

[42] Medeiros F.R., Lemos R.N.S.D., Ottati A.L.T., Araújo J.R.G., Machado K.K.G., Rodrigues A.A.C. (2009). Dinâmica Populacional Da Mosca-Negra-Dos-Citros *Aleurocanthus woglumi* Ashby (Hemiptera: Aleyrodidae) Em *Citrus* Spp. No Município De São Luís-Ma. Rev. Bras. Frutic..

[43] Reboita M.S., Gan M.A., Rocha R.P.d., Ambrizzi T. (2010). Regimes de Precipitação Na América Do Sul: Uma Revisão Bibliográfica. Rev. Bras. Meteorol..

[44] Phillips S.J., Dudík M. (2008). Modeling of Species Distributions with Maxent: New Extensions and a Comprehensive Evaluation. Ecography.

[45] Ji W., Gao G., Wei J. (2021). Potential Global Distribution of *Daktulosphaira vitifoliae* under Climate Change Based on Maxent. Insects.

[46] Wang C.-J., Wan J.-Z., Qu H., Zhang Z.-X. (2017). Modelling Plant Invasion Pathways in Protected Areas under Climate Change: Implication for Invasion Management. Web Ecol..

[47] Zou Y., Ge X., Guo S., Zhou Y., Wang T., Zong S. (2020). Impacts of Climate Change and Host Plant Availability on the Global Distribution of *Brontispa longissima* (Coleoptera: Chrysomelidae). Pest Manag. Sci..

[48] Cherry R., Fitzpatrick G. (1979). Intra-Tree Dispersion of Citrus Blackfly. Environ. Entomol..

[49] Quezada J. (1974). Biological Control of *Aleurocanthus woglumi* [Homoptera: Aleyrodidae] in El Salvador. Entomophaga.

[50] Silva A.B. (2005). Mosca negra dos citros, *Aleurocanthus woglumi* Ashby, praga potencial para a citricultura brasileira. Pragas e Doenças de Cultivos Amazônicos.

[51] Cunha M.d. (2003). Distribuição, Hospedeiros, Densidade Populacional, Aspectos Biológicos e Controle Químico da Mosca Negra dos Citros (*Aleurocanthus woglumi* Ashby) nas Condições do Estado do Pará. Master’s Thesis.

[52] Limberger L., Silva M.E.S. (2016). Precipitação Na Bacia Amazônica E Sua Associação À Variabilidade Da Temperatura Da Superfície Dos Oceanos Pacífico E Atlântico: Uma Revisão. GEOUSP Espaço Tempo (Online).

[53] Fisch G., Marengo J.A., Nobre C.A. (1996). Clima Da Amazônia. Climanálise-Boletim de Monitoramento e Análise Climática-Edição Comemorativa de.

[54] IBGE Produção Agrícola Municipal—PAM. https://www.ibge.gov.br/estatisticasnovoportal/economicas/agricul-turaepecuaria/9117producaoagricolamunicipalculturastemporariasepermanentes.html?edicao=18051&t=downloads.

[55] Phillips S.J., Dudík M., Elith J., Graham C.H., Lehmann A., Leathwick J., Ferrier S. (2009). Sample Selection Bias and Presence-Only Distribution Models: Implications for Background and Pseudo-Absence Data. Ecol. Appl..

[56] Zhao W., Hu A., Ni Z., Wang Q., Zhang E., Yang X., Dong H., Shen J., Zhu L., Wang J. (2019). Biodiversity Patterns across Taxonomic Groups along a Lake Water-Depth Gradient: Effects of Abiotic and Biotic Drivers. Sci. Total Environ..

